# Synthesis Route‐Controlled Atomic Ordering Optimizes Thermoelectric Transport in Bi_2_SbSe_3_

**DOI:** 10.1002/advs.78637

**Published:** 2026-11-09

**Authors:** Xiaoqian Wang, Shulin Bai, Huiqiang Liang, Rong Liu, Chao Liang, Yiqing Jia, Xiaokun Feng, Zhengguo Bai, Tian Gao, Huixin Pang, Yongjin Chen, Xin Qian, Zilu Wang, Haonan Shi, Sining Wang, Yi Wen, Peikang Bai, Lizhong Su, Li‐Dong Zhao

**Affiliations:** ^1^ School of Materials Science and Engineering Taiyuan University of Science and Technology Taiyuan China; ^2^ School of Materials Science and Engineering Beihang University Beijing China; ^3^ College of Physics Science and Technology Hebei University Baoding China; ^4^ Center for High Pressure Science and Technology Advanced Research (HPSTAR) Beijing China; ^5^ State Key Laboratory of Chemical Biology Shanghai Institute of Organic Chemistry University of Chinese Academy of Sciences Shanghai China; ^6^ State Key Laboratory of Advanced Stainless Steel Material Taiyuan China; ^7^ School of Materials Science and Technology China University of Geosciences (Beijing) Beijing China; ^8^ Shanxi Provincial Key Laboratory for Controlled Metal Solidification and Additive Manufacturing Taiyuan China

**Keywords:** atomic ordering, band structure, Bi_2_SbSe_3_, phonon transport, synthesis‐route engineering, thermoelectric materials

## Abstract

Thermoelectrics have leveraged band and phonon engineering to optimize performance, typically realized through chemical composition control via one‐pot doping or alloying. However, for compounds with complex atomic sites, the thermodynamic drive of conventional one‐pot synthesis generally favors a thermodynamically preferred atomic occupation, thereby limiting atomic ordering control and obscuring its influence on thermoelectric transport. Herein, we constructed a synthetic pathway for composition‐invariant band and phonon engineering via atomic ordering control in one‐third Sb‐alloyed BiSe (Bi_2_SbSe_3_), a compound with multiple crystallographically distinct cation sites. Using BiSbSe_3_ and Bi powders as precursors, we synthesized more disordered Bi_2_SbSe_3_ than the one‐pot synthesized counterpart, achieved by redistributing Sb atoms between the Bi_2_Se_3_ quintuple layers and the Bi bilayers in the BiSe structure. Reduced atomic ordering suppresses the higher‐lying conduction band contribution, lowering effective mass and enhancing carrier mobility, while simultaneously triggering lattice structure, slowing phonon velocities, and reducing lattice thermal conductivity. Finally, decreased atomic ordering enhances the *ZT* value of Bi_2_SbSe_3_ by ∼15% to ∼0.61 at 423 K, with an energy conversion efficiency of ∼4.3% under a temperature difference of ∼323 K. These results reveal that synthesis‐controlled atomic ordering is an effective strategy for enhancing thermoelectric performance in structurally complex materials.

## Graphical abstract

**Atomic ordering modulation enhancing the thermoelectric performance of Bi_2_SbSe_3_**. The solid‐state synthesis route redistributes Sb atoms between the Bi_2_Se_3_ quintuple layers and the Bi bilayers in the BiSe structure, resulting in a more disordered Bi_2_SbSe_3_ than the one‐pot synthesized counterpart. Reduced atomic ordering decreased conduction band degeneracy and intensified phonon scattering, thereby optimizing electron and phonon transport.

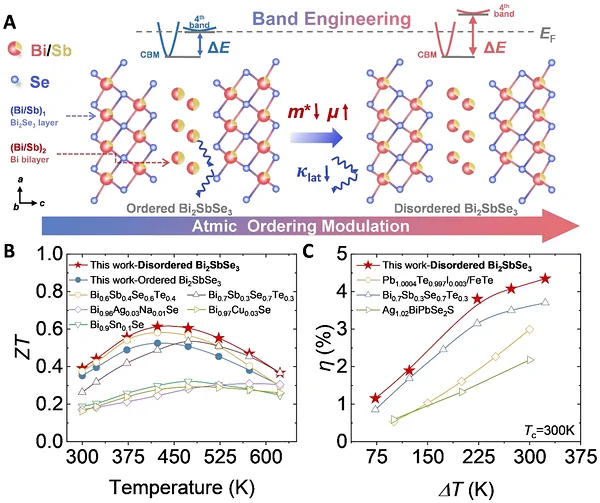

## 1 Introduction

Thermoelectric materials enable direct and reversible conversion between heat and electricity, offering immense potential for waste heat recovery, power generation, and refrigeration [1, 2, 3, 4]. The energy conversion efficiency *η* of thermoelectric materials is determined by the dimensionless figure of merit, *ZT* = *S* ^2^ *σT*/*κ*_tot_, where *S* is the Seebeck coefficient; *σ* is the electrical conductivity; *T* is the temperature in kelvin; and *κ*_tot_ is the total thermal conductivity, which includes the lattice thermal conductivity *κ*_lat_ and the electronic thermal conductivity *κ*_ele_ [5]. These parameters are intrinsically coupled, presenting a fundamental challenge in decoupling phonon and electron transport [6, 7, 8]. To address this, researchers have established material screening criteria that focus on intrinsically low *κ*_lat_ [9, 10, 11, 12] or on complex electronic band structures [13, 14]. Concurrently, physical mechanisms such as band/phonon engineering [15, 16, 17], lattice plainification [18, 19], and local lattice symmetry modulation [20, 21, 22, 23, 24, 25] have been deployed to decouple phonon‐electron transport [26].

In materials science implementation, these strategies have focused on microstructural and compositional control. Microstructure modification generally includes leveraging texturing and crystal growth to increase carrier mobility (*µ*) [20, 27], and designing multi‐scale grain architectures to suppress *κ*_lat_ concurrently while retaining *μ* [16, 17]. Underlying this, compositional tuning via doping or alloying remains the cornerstone of thermoelectric optimization in materials science practice. Typical operationalizations include introducing resonance levels or lattice plainification through trace element doping [18, 19], as well as defect engineering [28, 29, 30], promoting band divergence and lattice symmetry modulation via element alloying [20, 21, 22, 23, 24]. Conventionally, these compositional alterations are actualized using a one‐pot synthesis route. This entails heating stoichiometric raw materials above the melting point of the target compound until a homogeneous melt is achieved, followed by furnace cooling or quenching to retain the desired room‐temperature or high‐temperature thermodynamically stable phase. Adhering to this research paradigm has driven the rapid evolution of thermoelectrics over the last twenty years. This strategic focus has facilitated the discovery of new material families and delivered significant performance breakthroughs in many existing systems.

Notably, through compositional tuning, many excellent thermoelectric materials have evolved into multicomponent compounds. This has introduced another materials‐science perspective on decoupling phonon‐electron transport: designing atomic arrangements to control atomic ordering [31]. Alloying foreign elements results in crystallographic sites that can be occupied by different elements, leading to potentially diverse atomic arrangements. Since band structures and phonon dispersions depend on both chemical composition and coordination, modifying atomic site configuration offers a unique way to fully exploit the thermoelectric potential of multicomponent materials. The strategy has been demonstrated in AgSbTe_2_, where Cd doping altered the atomic ordering of the nearly randomly occupied Ag/Sb cation sublattice, leading to an optimized electronic structure and a maximum *ZT* of ∼2.6 [32]. Unlike this composition‐driven ordering modulation, some materials possess intrinsically complex atomic occupations arising from their crystal structures, enabling atomic ordering control without altering the chemical composition. BiSe is one representative example.

BiSe is a member of the homologous series (Bi_2_)_m_(Bi_2_Se_3_)_n_ (where m:n = 1:2) with intrinsically low lattice thermal conductivity, showing thermoelectric potential due to its complex crystal structure [33, 34]. BiSe exhibits a layered structure, comprising a bismuth bilayer (Bi_2_) sandwiched between two Bi_2_Se_3_ quintuple layers. Compared with Bi_2_Se_3_, the ultralow lattice thermal conductivity in BiSe originates from localized vibrations within Bi bilayers, which generate low‐frequency optical phonon branches. The strong coupling between these optical modes and acoustic phonons suppresses the cutoff frequencies of the acoustic phonon branches, thereby reducing the mean free path [12]. Based on the resulting ultralow lattice thermal conductivity, the thermoelectric performance of BiSe can be further enhanced through compositional tuning via alloying with foreign elements, particularly by substituting Sb for Bi. This optimizes carrier concentration (*n*) to enhance electrical properties while introducing point defects to scatter phonons. Notably, BiSe cation sites can be broadly categorized into two types: one in the Bi_2_Se_3_ quintuple layers, marked as (Bi/Sb)_1_ sites, and the other in the Bi bilayer, marked as (Bi/Sb)_2_ sites (Figure 1A). This indicates that Sb atoms can occupy either site, and the ratio of Sb atoms at the different sites affects the degree of atomic ordering. Given the elemental disparity between Sb and Bi and the distinct coordination environment of the cationic sites within the lattice, the difference in atomic arrangements could hold profound impacts on the thermoelectric performance of Sb‐alloyed BiSe with identical compositions.

**FIGURE 1:**
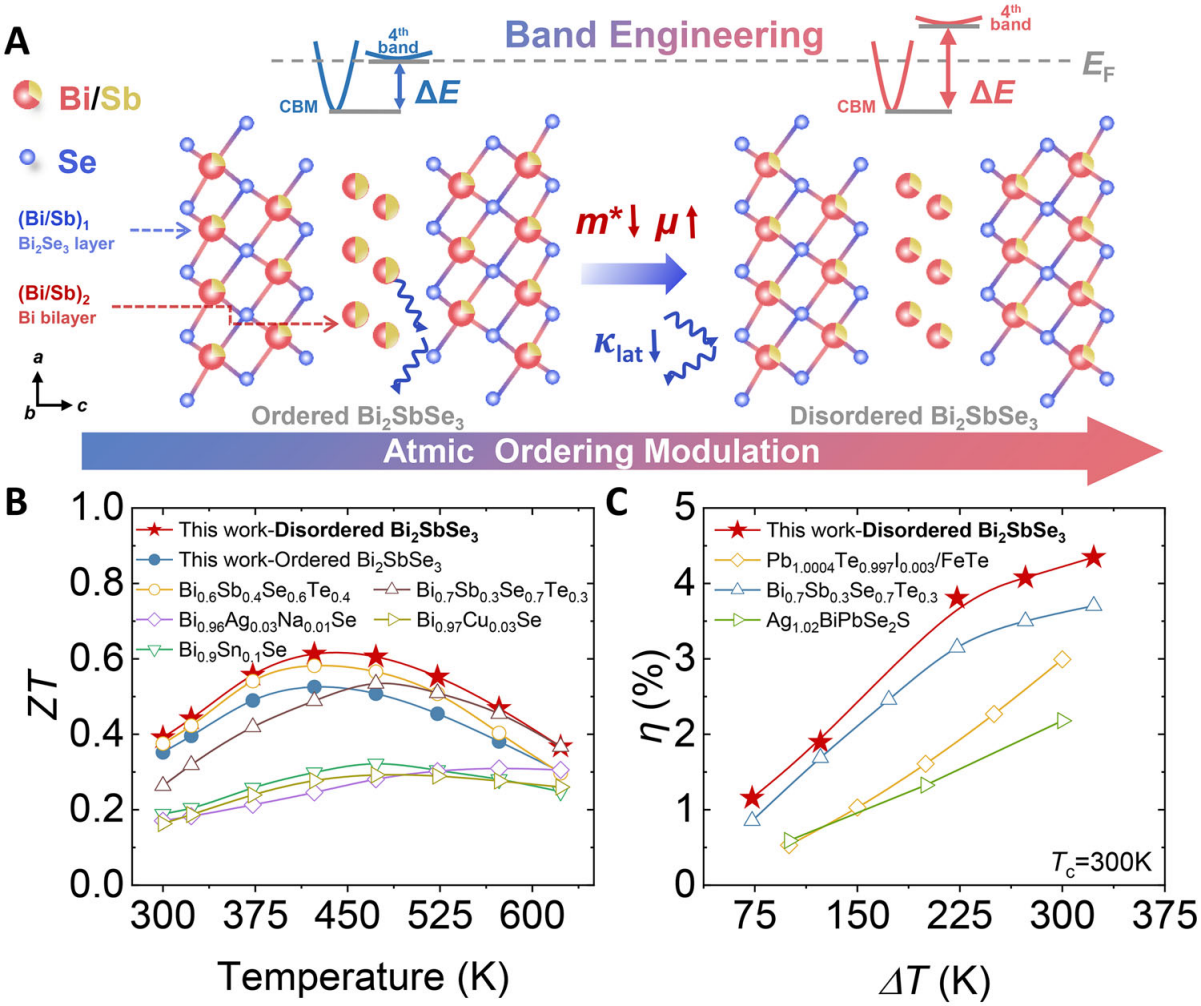
Atomic ordering modulation enhancing the thermoelectric performance of Bi_2_SbSe_3_. (A) The solid‐state synthesis route redistributes Sb atoms between the Bi_2_Se_3_ quintuple layers and the Bi bilayers in the BiSe structure, resulting in a more disordered Bi_2_SbSe_3_ than the one‐pot synthesized counterpart. Reduced atomic ordering decreased conduction band degeneracy and intensified phonon scattering, thereby optimizing electron and phonon transport. (B) Comparison of *ZT* between this work and reported n‐type BiSe‐based polycrystals [35, 36, 37, 38, 39]. (C) Comparison of single‐leg thermoelectric conversion efficiency between this work and other reported TE materials [39, 40, 41].

Atomic ordering holds substantial promise for further optimizing the thermoelectric performance of BiSe. However, to date, the effect of atomic ordering on the thermoelectric transport of Sb‐alloyed BiSe has not been thoroughly investigated. A key challenge in such research is developing viable methods to manipulate the atomic arrangements. As with most thermoelectric materials, BiSe relies on the one‐pot synthesis as its main fabrication method. Since the one‐pot synthesis route naturally seeks the most thermodynamically stable product, it fixes the atomic arrangements to the lowest‐energy configuration. Although methods like vapor deposition theoretically control atomic arrangements in different layers, their inefficiency makes them unsuitable for bulk thermoelectric. This drives us to pursue atomic ordering by modifying the synthesis route and to experimentally explore its effect on thermoelectric transport properties.

In this work, we developed a synthetic route for Sb‐alloyed BiSe that manipulates atomic ordering and optimizes thermoelectric performance. Bi_2_SbSe_3_ was selected as the optimal composition based on preliminary screening of Sb‐alloyed BiSe samples (Figures S1–S3). Characterization confirmed that the one‐pot‐synthesized Bi_2_SbSe_3_ exhibited an ordered cation distribution (ordered Bi_2_SbSe_3_). Notably, we discovered that a solid‐state reaction using BiSbSe_3_ and Bi as precursors also yielded Bi_2_SbSe_3_, but with a distinct Bi/Sb distribution. Compared with the ordered Bi_2_SbSe_3_ obtained by one‐pot synthesis. The solid‐state route preferentially promoted Sb occupation at the Bi_2_Se_3_ layer sites, thus favoring the formation of a disordered Bi_2_SbSe_3_ configuration. The reduced atomic ordering decreased the conduction band degeneracy, thereby reducing the effective mass (m*) and enhancing carrier mobility (*μ*). Concurrently, the site preference of Sb atoms affected the lattice structure, inducing pronounced lattice modification that suppresses phonon group velocity and thus reduces lattice thermal conductivity. Finally, a peak *ZT* of ∼0.61 was achieved at 423 K in disordered Bi_2_SbSe_3_, representing a ∼15% improvement over the ordered counterpart synthesized via the one‑pot method (Figure 1B). Moreover, a single‐leg conversion efficiency (*η*) of ∼4.3% at 300–623 K demonstrated the power generation potential of disordered Bi_2_SbSe_3_ (Figure 1C). These results demonstrate that synthesis route‐controlled atomic ordering is a viable strategy for optimizing thermoelectric performance, providing fresh perspectives for other thermoelectric materials with complex atomic site preferences.

## 2 Results

Ordered Bi_2_SbSe_3_ was synthesized via a one‐pot method, whereas the disordered counterpart was prepared via a solid‐state reaction and subjected to repeated spark plasma sintering (SPS) deformation to achieve a crystallographic texture comparable to that of the ordered counterpart (Figures S4–S7). The thermoelectric properties of both samples presented in the main text were measured parallel to the SPS pressing direction. The comparable preferred orientations were confirmed by XRD analysis and similar Lotgering factors (Figure S8), thereby minimizing the influence of crystallographic texture on the comparison of thermoelectric transport properties.

### 2.1 Synthesis Route‐Controlled Atomic Ordering

The crystal structures of ordered and disordered Bi_2_SbSe_3_ were first examined by SR‐XRD. Both samples could be indexed to the BiSe phase belonging to space group $P\overset{-}{3}m1$ (PDF#29–0246) (Figure 2A), with no detectable impurity phases, confirming their identical phase compositions. SEM‐EDS analysis further showed uniform distributions of Bi, Sb, and Se in both samples, with highly consistent average compositions and no obvious elemental segregation (Figures S9 and S10).

**FIGURE 2:**
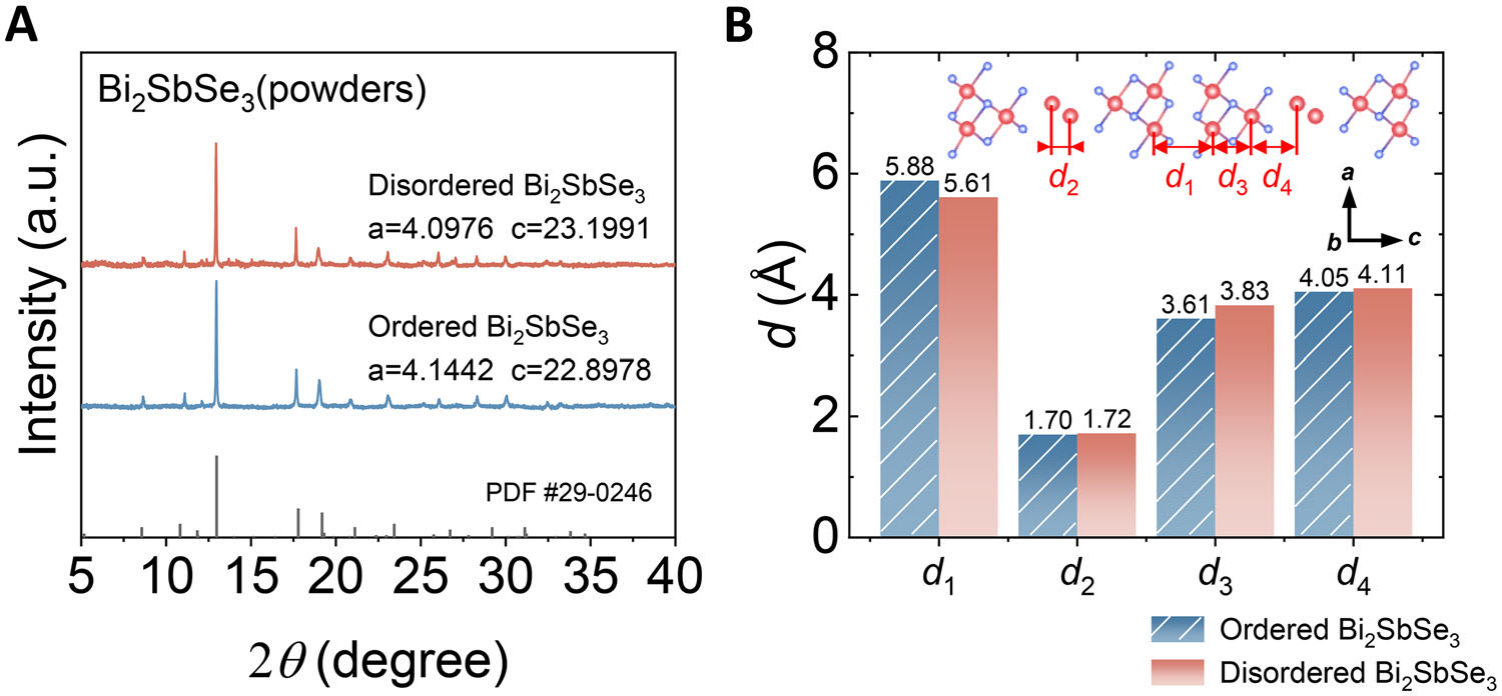
X‐ray diffraction results. (A) Synchrotron radiation X‐ray diffraction (SR‐XRD) patterns of ordered Bi_2_SbSe_3_ and disordered Bi_2_SbSe_3_, with refined lattice parameters indicated. (B) Comparison of interatomic distances obtained from Rietveld refinement.

To clarify the structural evolution associated with the distinct atomic‐ordering states produced by different synthesis routes, SR‐XRD refinement was carried out to precisely extract the lattice parameters. The refinement results revealed systematic variations in the lattice parameters and interatomic distances between the two samples (Figure 2B). Specifically, the *a*‐axis lattice parameter of disordered Bi_2_SbSe_3_ slightly decreased, whereas the *c*‐axis lattice parameter increased, accompanied by an overall expansion of Bi‐Bi interatomic distances within the unit cell, suggesting enlarged interlayer spacing. The SR‐XRD results indicate that atomic ordering may be regulated by the synthesis route without altering the phase composition or crystallographic texture. High‐temperature SR‐XRD measurements further revealed no obvious structural transition in disordered Bi_2_SbSe_3_ up to 523 K (Figure S11), indicating good structural stability within this temperature range.

To further elucidate the synthesis route‐controlled atomic‐ordering states, X‐ray photoelectron spectroscopy (XPS) measurements were performed to probe the atomic‐site occupancy based on the distinct chemical states of Bi/Sb in Bi_2_SbSe_3_ (Figure 3A,B). In the crystal structure of Bi_2_SbSe_3_, Bi/Sb atoms occupying the Bi_2_Se_3_ quintuple layers exist as Bi^3+^/Sb^3+^, whereas those located in the metallic bilayers exist as Bi^0^/Sb^0^. Both ordered and disordered Bi_2_SbSe_3_ exhibited characteristic Sb 3d doublets corresponding to Sb 3d_5/2_ and Sb 3d_3/2_, indicating the coexistence of Sb^3+^ and Sb^0^ species. For a totally disordered Bi_2_SbSe_3_ structure, random Bi/Sb occupation results in a theoretical random Sb occupancy of one‐third (33.3%) statistically in the metallic bilayer. Notably, XPS revealed distinct Sb^3+^/Sb^0^ ratios for the two samples. The Sb^0^ occupancies of the one‐pot‐synthesized and solid‐state‐reacted samples were ∼52.82% and ∼42.30%, respectively, deviating from the theoretical random value by ∼20 and ∼9 percentage points. The larger deviation suggested a higher degree of atomic ordering in the one‐pot‐synthesized sample, whereas the smaller deviation suggested a distribution closer to the random‐occupation state in the solid‐state‐reacted sample. The different atomic‐ordering states may originate from the distinct thermal histories and associated kinetics of atomic diffusion in the two synthesis routes. The high reaction temperature (1123 K) and subsequent annealing at 893 K during one‐pot synthesis may provide greater kinetic access for atomic diffusion and site redistribution [42, 43]. In contrast, the lower temperature (773 K) used for the solid‐state reaction may limit long‐range atomic diffusion and site redistribution, such that more nonequilibrium site occupations could be retained and result in a lower degree of atomic ordering.

**FIGURE 3:**
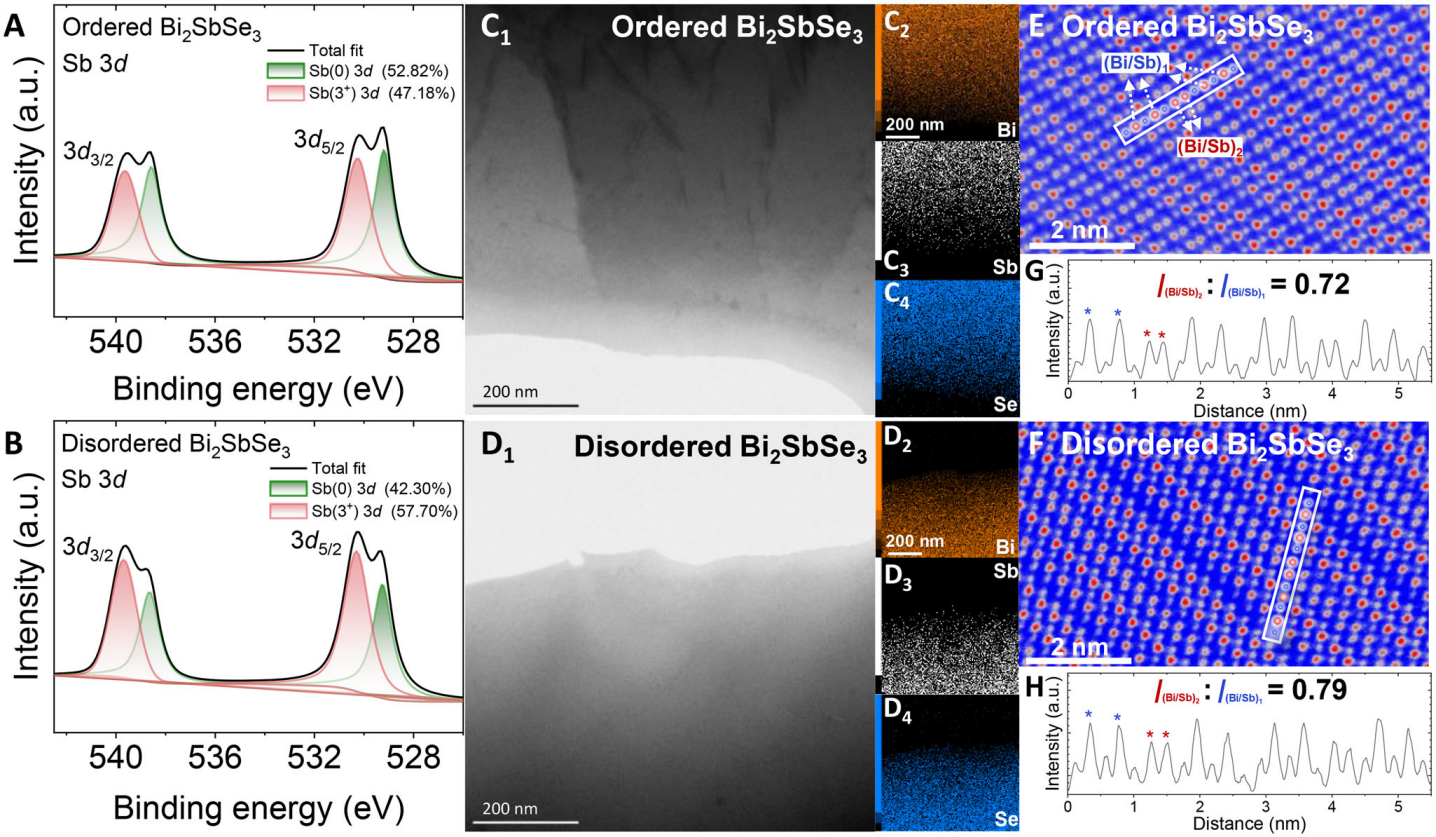
Atomic ordering characterization. X‐ray photoelectron spectroscopy (XPS) results of the Sb element for (A) ordered Bi_2_SbSe_3_ and (B) disordered Bi_2_SbSe_3_. Annular bright‐field scanning transmission electron microscopy (ABF‐STEM) images for (C) ordered Bi_2_SbSe_3_ and (D) disordered Bi_2_SbSe_3_, with corresponding energy‐dispersive spectroscopy (EDS) elemental maps of Bi, Sb, and Se. Higher‐resolution annular dark‐field (ADF) STEM images for (E) ordered Bi_2_SbSe_3_ and (F) disordered Bi_2_SbSe_3_. Line‐scan intensity profiles for (G) ordered Bi_2_SbSe_3_ and (H) disordered Bi_2_SbSe_3_, revealing differences in Sb site‐occupancy preferences.

The atomic‐ordering states were further directly visualized by aberration‐corrected scanning transmission electron microscopy (STEM). Annular bright‐field STEM (ABF‐STEM) imaging and energy dispersive spectroscopy (EDS) elemental mapping indicated a homogeneous elemental distribution in both ordered and disordered Bi_2_SbSe_3_ (Figure 3C,D). High‐angle annular dark‐field STEM (ADF‐STEM) images clearly resolved the characteristic layered structure consisting of quintuple layers separated by metallic bilayers (Figure 3E,F).

The relative HAADF‐STEM intensities of the quintuple layers and metallic bilayers were quantified from the boxed regions in Figure 3E,F, and representative intensity line profiles were shown in Figure 3G,H. Because the HAADF‐STEM intensity is approximately proportional to the atomic number (Z‐contrast), the intensity difference between the two sublattices directly reflects the relative Bi/Sb occupation. We defined the cation sites in the quintuple layers as (Bi/Sb)_1_, and those in the metallic bilayer as (Bi/Sb)_2_. The relative intensity ratios at the (Bi/Sb)_2_ and (Bi/Sb)_1_ sites were 0.72 and 0.79 for ordered and disordered Bi_2_SbSe_3_, respectively (Table S2). Since Bi has a higher atomic number than Sb, the more Bi occupies a given atomic site, the stronger its intensity appears in the HAADF‐STEM image. Disordered Bi_2_SbSe_3_ shows a higher value of intensity ratio at the (Bi/Sb)_2_ and (Bi/Sb)_1_ sites, indicating a higher Bi fraction in the metallic bilayers than that of ordered Bi_2_SbSe_3_, i.e., less Sb in the metallic bilayer. Together, the XPS and STEM results provided direct experimental evidence that different synthesis routes produce distinct atomic‐ordering states in Bi_2_SbSe_3_.

### 2.2 Electrical Transport Performance

Bi_2_SbSe_3_ exhibited anisotropic thermoelectric transport properties owing to its layered crystal structure (Figures S2, S3, and S5–S7). The following discussion focused on the transport properties measured parallel to the SPS pressure direction (SPS*//*), which exhibited superior thermoelectric performance. The influence of atomic ordering on the electrical transport properties was first evaluated. Compared with ordered Bi_2_SbSe_3_, disordered Bi_2_SbSe_3_ exhibited an obviously enhanced *σ* over the entire temperature range from 300 to 623 K (Figure 4A). The negative *S* values indicated that both samples were n‐type semiconductors (Figure 4B). With increasing temperature, |*S*| initially increased and subsequently decreased due to the onset of bipolar diffusion effect at elevated temperatures. Meanwhile, disordered Bi_2_SbSe_3_ exhibits reduced |*S*| compared with ordered Bi_2_SbSe_3_. In the disordered Bi_2_SbSe_3_, simultaneous increase in *σ* outweighed the reduction in |*S*|, yielding an improved power factor (*PF*). This disordered sample exhibited a markedly higher *PF* across 300–623 K (Figure 4C), reaching ∼9.9 µW cm^−1^ K^−2^ at room temperature.

**FIGURE 4:**
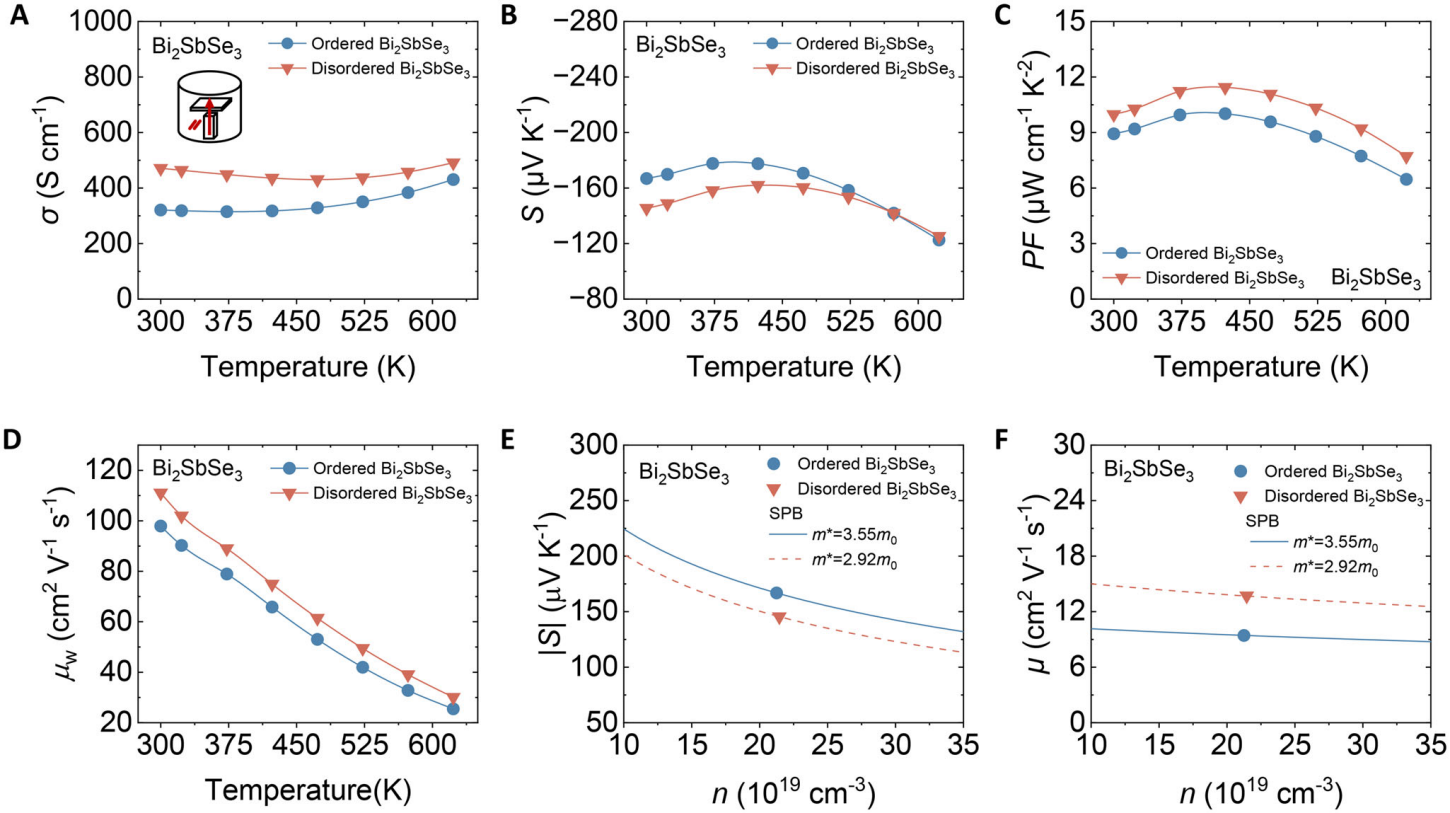
Electrical transport properties of ordered Bi_2_SbSe_3_ and disordered Bi_2_SbSe_3_. (A) Electrical conductivity (*σ*). (B) Seebeck coefficient (*S*). (C) Power factor (*PF*). (D) Weighted mobility (*μ*_w_) as a function of temperature. (E) *S* as a function of carrier concentration at 300 K. (F) Carrier mobility (*μ*) as a function of carrier concentration at 300 K.

To uncover the origin of the improved electrical transport, the weighted mobility (*μ*_w_) was evaluated (μ_*w*_ = μ(m*)^3/2^) [44]. As a transport descriptor that incorporates both *μ* and *m* ^*^, *μ*_w_ provides insight into the coupling between the electronic structure and carrier transport. Disordered Bi_2_SbSe_3_ exhibited consistently higher *μ*_w_ than ordered Bi_2_SbSe_3_ over the entire temperature range (Figure 4D), indicating an optimized balance between m* and *μ*.

To further elucidate the carrier transport mechanism underlying the enhanced electrical transport performance, the room‐temperature Pisarenko and Hall analyses were carried out. The Pisarenko plot fitted using the single parabolic band (SPB) model showed that ordered Bi_2_SbSe_3_ approximately followed the theoretical line (m* = 3.55*m*_0_), whereas disordered Bi_2_SbSe_3_ corresponded to a reduced m* of 2.92*m*_0_ (Figure 4E). The reduced m* accounted for the lower *S* of disordered Bi_2_SbSe_3_. Meanwhile, both samples exhibited nearly identical *n* (∼2.1 × 10^20^ cm^−3^), while disordered Bi_2_SbSe_3_ possessed markedly higher *μ* (Figure 4F). These results indicated that the enhanced *σ* predominantly originated from improved *μ* rather than changes in *n*. To further rule out the possible influence of grain growth induced by repeated SPS processing on carrier mobility in disordered Bi_2_SbSe_3_, grain size characterization was performed using electron backscatter diffraction (EBSD). The average grain sizes of ordered and disordered Bi_2_SbSe_3_ were approximately 3.1 and 3.9 µm, respectively, showing a relatively modest difference (Figures S13 and S14). To assess the contribution of grain‐boundary scattering, the electron mean free paths (*l*_e_) were estimated to be approximately 1.15 nm (ordered) and 1.67 nm (disordered). Both values were much smaller than the corresponding grain sizes, suggesting that grain‐boundary scattering was unlikely to dominate electrical transport. Accordingly, the enhanced electrical transport may stem from deeper intrinsic effects associated with the distinct atomic‐ordering states.

The electronic band structures of ordered and disordered Bi_2_SbSe_3_ were therefore investigated by density functional theory (DFT) calculations. The calculated results revealed that changes in atomic ordering redistributed the band structure (Figure 5A,B). Specifically, disordered Bi_2_SbSe_3_ displayed a markedly larger energy separation (Δ*E*) between the conduction band minimum (CBM) and the fourth conduction band (heavy band) than the ordered sample, and the extracted Δ*E* values further confirmed this difference (Figure 5C). In ordered Bi_2_SbSe_3_, the small Δ*E* positioned the Fermi level (*E*_F_) at the edge of the fourth conduction band, allowing this heavy band to participate in carrier transport. By contrast, in disordered Bi_2_SbSe_3_, the heavy conduction band was shifted above the *E*_F_ and therefore no longer contributed to carrier transport. This conduction‐band redistribution was further supported by the decreased projected density of states (PDOS) near *E*_F_ in disordered Bi_2_SbSe_3_ (Figure S12). As a consequence, the number of conducting valleys reduced from four to three in disordered Bi_2_SbSe_3_. This reduced band degeneracy would lower m*and thereby enhance *μ*, which was in good agreement with the experimental observations. Overall, these results demonstrated that the distinct atomic‐ordering states established by different synthesis routes modulated the conduction‐band structure, leading to superior carrier transport in disordered Bi_2_SbSe_3_.

**FIGURE 5:**
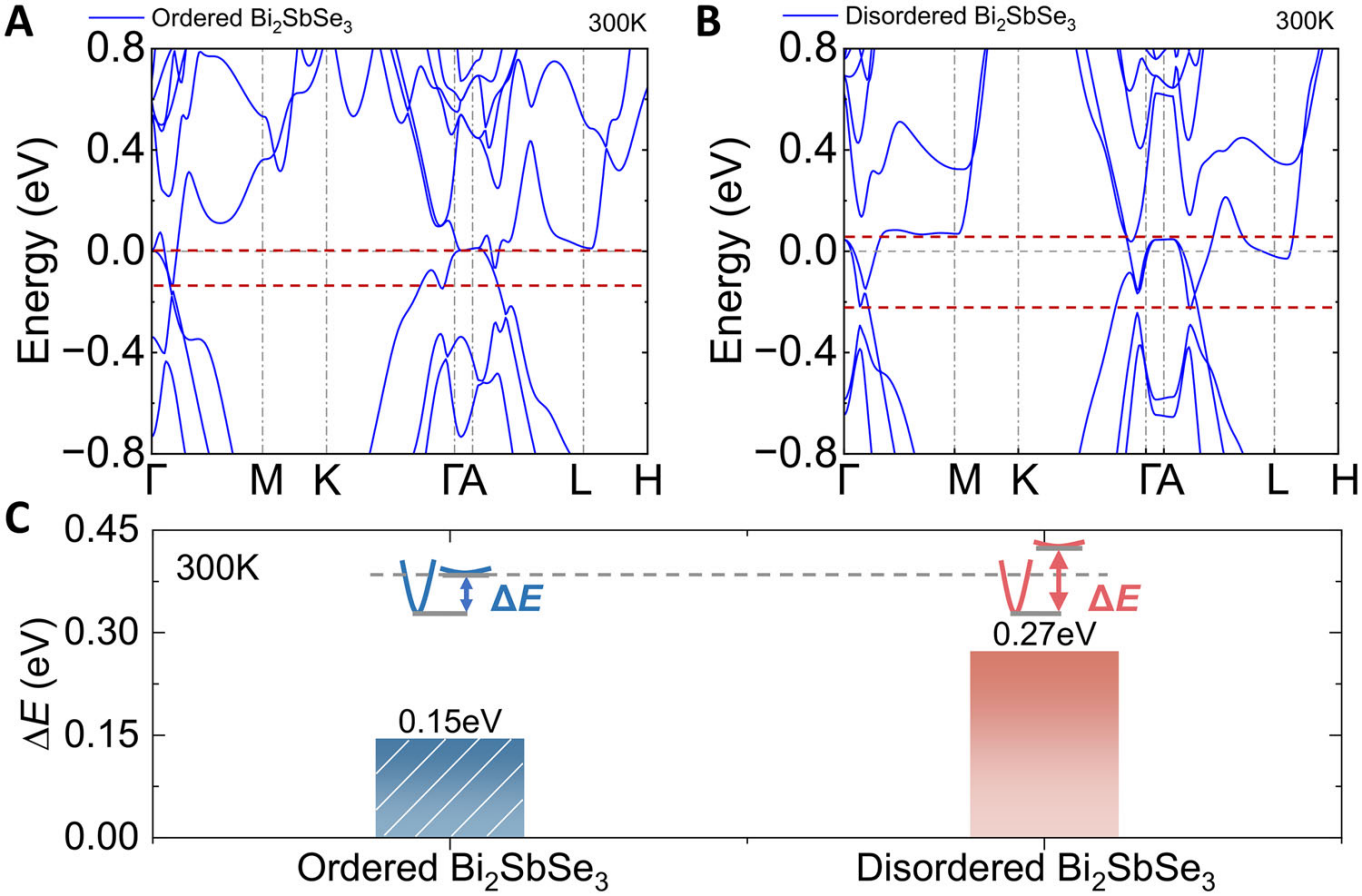
Band structure. The calculated electronic band structures of (A) ordered and (B) disordered Bi_2_SbSe_3_ based on SR‐XRD refinement data. Disordered Bi_2_SbSe_3_ shows a remarkably enlarged energy separation (Δ*E*) between the conduction band minimum (CBM) and the fourth conduction band, indicating the divergence of the conduction bands. (C) Quantitative comparison of the extracted Δ*E* values for ordered and disordered Bi_2_SbSe_3_.

### 2.3 Thermal Transport Properties and Device Performance

Besides the optimized electrical transport, atomic ordering also significantly influenced the thermal transport behavior of Bi_2_SbSe_3_. Although disordered Bi_2_SbSe_3_ exhibited a higher *κ*_ele_ owing to its enhanced *σ* (Figure S15A), its *κ*_tot_ remained slightly lower than that of ordered Bi_2_SbSe_3_ over the entire temperature range (Figure 6A). This result indicated that the increase in *κ*_ele_ was outweighed by a more pronounced reduction in *κ*_lat_.

**FIGURE 6:**
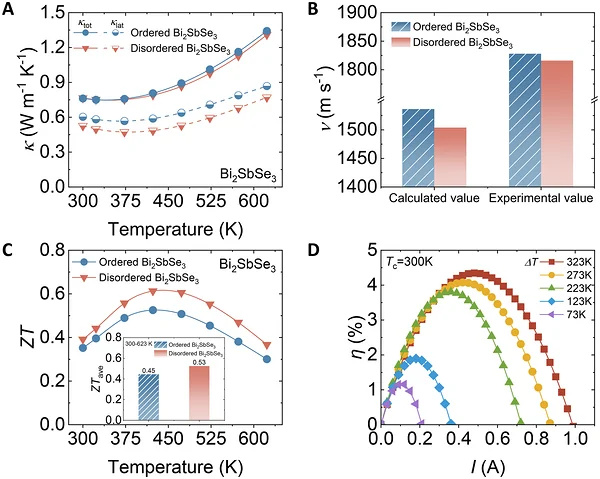
Thermal transport properties and thermoelectric performance of ordered Bi_2_SbSe_3_ and disordered Bi_2_SbSe_3_. (A) Total thermal conductivity (*κ*_tot_) and lattice thermal conductivity (*κ*_lat_). (B) Experimental and calculated sound velocities (*v*). (C) *ZT* values, with the inset showing the average *ZT* (*ZT*_ave_). (D) Measured conversion efficiency (*η*) for disordered Bi_2_SbSe_3_ single‐leg device as a function of current *I* at different hot‐side temperatures (*T*_h_), with the cold end temperature (*T*_c_) fixed at ∼300 K.

To elucidate the origin of the reduced *κ*_lat_ in disordered Bi_2_SbSe_3_, the possible contribution from grain‐size effects was first assessed. The disordered sample possessed a slightly larger average grain size (Figures S13 and S14), which tends to weaken grain‐boundary phonon scattering. Furthermore, kernel average misorientation mapping (KAM in Figures S13C and S14C) and ABF‐STEM images (Figure 3C_1_,D_1_) revealed lower local lattice misorientation and fewer dislocations and lattice‐strain defects in disordered Bi_2_SbSe_3_. These microstructure features would generally favor higher *κ*_lat_, suggesting that other mechanisms are responsible for its reduction in *κ*_lat_. Accordingly, the effect of Bi/Sb site disorder on phonon transport was further considered. The increased Bi/Sb site disorder can introduce local mass fluctuations and force‐constant perturbations, which are expected to enhance phonon scattering and thereby contribute to the reduced *κ*_lat_ [45]. Meanwhile, the redistribution of Bi/Sb among different atomic sites is accompanied by pronounced structural changes. Compared with ordered Bi_2_SbSe_3_, disordered Bi_2_SbSe_3_ exhibits a higher relative Bi occupancy in the metallic bilayers, together with enlarged interlayer spacing (Figure 2B). These changes in site occupancy and structure may modify the local bonding environment and interlayer interactions, thereby facilitating lattice softening. In addition to enhanced phonon scattering, the resulting structural evolution can reduce the phonon propagation velocity (*v*), further suppressing lattice heat transport [12, 46]. To examine this effect, *v* was evaluated through both experimental measurements and theoretical calculations (Tables S3 and S4). Both approaches consistently showed lower *v* for disordered Bi_2_SbSe_3_ (Figure 6B), providing further evidence for its suppressed phonon transport [47] and the reduced *κ*_lat_.

The quality factor (*B*), which combines the contributions of *µ*_w_ and *κ*_lat_, served as a descriptor of the intrinsic thermoelectric potential (Figure S16) [44, 48]. Disordered Bi_2_SbSe_3_ maintained a consistently higher *B* over the entire temperature range, indicating that the synergistic optimization of electrical and thermal transport enhanced its intrinsic thermoelectric performance. As a result, disordered Bi_2_SbSe_3_ exhibited higher *ZT* values than ordered Bi_2_SbSe_3_ throughout the entire temperature range, reaching a maximum *ZT* of ∼0.61 at 423 K (Figure 6C) and a higher average *ZT* (*ZT*_ave_) (Figure 6C **inset**). To validate the thermoelectric performance of the optimized material and assess its device potential, a single‐leg thermoelectric device based on disordered Bi_2_SbSe_3_ was fabricated. The device delivered a peak power output (*P*) of ∼10 mW (Figure S17) and a maximum *η* of 4.3% (Figure 6D) at a current of 0.48 A under a temperature difference (Δ*T*) of 323 K. The experimental conversion efficiency is lower than the theoretical prediction (Figure S18A). The specific contact resistivity is approximately 189 µΩ cm^2^ at room temperature, corresponding to 2.1 mΩ per interface (Figure S18B). The resulting interfacial electrical loss, together with thermal contact resistance and parasitic heat losses [49], may contribute to the efficiency discrepancy. Even without further doping optimization, the promising device performance (Figure 1C) highlighted the potential of synthesis‐route‐controlled atomic ordering for further thermoelectric performance enhancement. Considering the importance of thermoelectric stability for practical energy‐conversion applications [50, 51], the electrical transport stability of disordered Bi_2_SbSe_3_ was further evaluated by temperature‐cycling measurements (with maximum temperatures of 523, 573, and 623 K) following thermoelectric transport measurements up to 623 K (Figure S19).

Good transport reversibility was observed at lower cycling temperatures (523 and 573 K), supporting its thermoelectric application potential within the temperature range of peak thermoelectric performance. Irreversible deviations emerged after cycling to 623 K, suggesting possible relaxation of the kinetically stabilized metastable atomic configuration and highlighting the importance of operating‐temperature control.

## 3 Conclusions

In summary, we demonstrate that synthesis route‐controlled atomic ordering can serve as an effective strategy to optimize the thermoelectric transport in Sb‐alloyed BiSe. By using pre‐synthesized BiSbSe_3_ and Bi powders as precursors, a more atomically disordered Bi_2_SbSe_3_ was obtained through a solid‐state reaction, in which the atomic‐ordering state was modulated through the redistribution of Sb atoms between Bi_2_Se_3_ quintuple layers and the metallic Bi bilayers. The resulting reduction in atomic ordering reconstructs the electronic structure, enlarging the energy separation between the CBM and the fourth conduction band. As a result, the originally closed conduction bands undergo divergence, which reduces m* and enhances *µ*, leading to improved electrical transport. Meanwhile, the atomic‐ordering modulation enlarged the interlayer spacing and lowered the sound velocity, giving rise to a further reduction in *κ*_lat_. Owing to the simultaneous improvement in carrier transport and suppression of lattice heat transport, disordered Bi_2_SbSe_3_ realizes a maximum *ZT* of ∼0.61 at 423 K, achieving a ∼15% increase compared with the one‐pot synthesized ordered counterpart. A single‐leg device based on disordered Bi_2_SbSe_3_ further delivers a maximum *η* of 4.3% under a Δ*T* of 323 K, indicating its potential for thermoelectric power generation. These findings highlight atomic ordering controlled by synthesis route as an effective strategy for decoupling electron and phonon transport in structurally complex thermoelectric materials. More broadly, the synthesis‐route‐controlled atomic‐ordering strategy may potentially be extended to other thermoelectric systems with multiple nonequivalent crystallographic sites and distinct elemental site preferences. For example, the Bi_2_Te_3_–Bi_2_Se_3_ alloy system and type‐I clathrates possess such site‐occupation characteristics [52, 53]. These crystallographic features may provide an additional degree of freedom for regulating atomic distributions and ordering states through synthesis‐route control. Meanwhile, given that kinetically stabilized atomic‐ordering states are thermodynamically metastable, appropriate operating temperatures are essential for retaining these states. Taken together, the synthesis‐route‐controlled atomic ordering raised in this work may provide a practical approach to modulating electron and phonon transport, thereby further enhancing the thermoelectric performance of conventional materials.

## 4 Methods

### 4.1 Sample Preparation

A series of Bi_1‐x_Sb_x_Se samples were first synthesized via a one‐pot melting route. Stoichiometric Bi, Sb, and Se (99.999%, Hebei Jiuyue, China) were sealed in evacuated quartz tubes and subjected to a multistep heating program for synthesis, including heating to 723 K over 12 h, further ramping to 1123 K over 5 h, holding for 6 h, annealing at 893 K for 6 h, followed by furnace cooling to room temperature. The obtained bulks were grounded and densified by spark plasma sintering (SPS, SPS‐2T‐20‐mini, Nanjing Boyuntong Instrument, China) under identical conditions (723 K, 50 MPa), from which Bi_2_SbSe_3_ was identified as the optimal composition and referred to as the “ordered” Bi_2_SbSe_3_.

A solid‐state reaction route was developed to obtain a structurally disordered Bi_2_SbSe_3_ counterpart at the same composition. The BiSbSe_3_ precursor powders, prepared according to a reported method [12], were mechanically mixed with Bi powder at a 1:1 molar ratio, resealed in evacuated quartz tubes, heated to 773 K, held for 12 h, and furnace cooled to room temperature. The resulting powders were subjected to repeated SPS consolidation and deformation at 723 K under 50 MPa using graphite dies with progressively increasing diameters (10, 12, and 15 mm) to enhance its preferred orientation (Figure S4) to a crystallographic texture comparable to that of the ordered counterpart. The final deformed sample was hereafter referred to as “disordered Bi_2_SbSe_3_”. Overall, we called one‐pot synthesized Bi_2_SbSe_3_ as ordered Bi_2_SbSe_3_, and solid‐state reaction synthesized Bi_2_SbSe_3_ as disordered Bi_2_SbSe_3_ in this work.

### 4.2 X‐Ray Diffraction

The phase composition and orientation of the samples were characterized by X‐ray diffraction (XRD, FRINGE CLASS SY X‐ray, LANScientific, China) using Cu Kα radiation in reflection geometry, operated at 30 kV and 16 mA. For detailed structural analysis, high‐resolution synchrotron radiation X‐ray diffraction (SR‐XRD) measurements were performed at the BL14B1 beamline of the Shanghai Synchrotron Radiation Facility (SSRF) using X‐rays with a wavelength of 0.6887 Å [54]. The SR‐XRD data were analyzed using GSAS‐II to determine the lattice parameters and interatomic distances.

### 4.3 X‐Ray Photoelectron Spectroscopy

Elemental valence states were determined using X‐ray photoelectron spectroscopy (XPS, ESCALAB 250Xi, Thermo Fisher Scientific Inc., USA) with an Al Kα radiation source (1486.6 eV photon energy, 300 W).

### 4.4 Scanning Transmission Electron Microscopy and Elemental Analysis

The scanning transmission electron microscopy (STEM) and energy dispersive spectroscopy (EDS) analyses were carried out by JEM F200 with a Hitachi HF 5000 cold field gun at 200 kV.

### 4.5 Electron Backscatter Diffraction

Electron backscatter diffraction (EBSD) characterization was performed using a field‐emission scanning electron microscope (SEM4000X, CIQTEK, China) equipped with an EBSD detector (C‐SWIFT, Oxford Instruments).

### 4.6 Electrical Transport

The obtained samples were cut into 3 × 3 × 8 mm^3^ blocks parallel and perpendicular to the SPS direction. Electrical conductivity (*σ*) and Seebeck coefficient (*S*) were simultaneously measured using a thermoelectric measurement system (CTA 3, Cryoall, China) in a helium atmosphere. The uncertainties for both *σ* and *S* were within 5%.

### 4.7 Hall Measurement

Samples were cut into slices approximately 6 × 6 × 1 mm^3^. The Hall coefficient was measured at 300 K under a reversible magnetic field of 0.9 T using a Hall measurement system (CH 500, Beijing Cuihai Jiacheng Magnetoelectric Technology Co., China). Carrier concentration was calculated through *n* = 1/(*eR*_H_), and carrier mobility was determined via *µ* = *σR*_H_.

### 4.8 Thermal Conductivity

The samples were cut into 6 × 6 × 1 mm^3^ slices in both parallel and perpendicular directions for measurements. The overall thermal conductivity was calculated by κ_*tot*_  =  ρ*DC_p_*, where *D* was measured using a thermal conductivity analyzer (CLA 1000, Cryoall, China) (Figure S15B), the density (*ρ*) of samples is derived from Archimedes' method (Table S1), and the heat capacity (*C*_p_) was estimated as 0.1927 J g^−1^ K^−1^, based on the Dulong Petit law using the relation *C*_p_ = 3*R*/*M*. The electronic thermal conductivity is calculated based on the Wiedemann‐Franz relation κ_*ele*_  =  *L*σ*T*, where *L* is the Lorenz number estimated using the single parabolic band (SPB) model (Equations S1–S3), and *T* is the absolute temperature.

### 4.9 Contact Resistance Measurement

The contact resistance was evaluated using homemade equipment. A constant current of 50 mA was provided by a power source connected to the contact ends of the sample. The voltage variation across the thermoelectric material/contact interface was recorded at the sample surface using a scanning probe. The contact resistivity (*ρ*_c_) was calculated as *ρ*_c_ = (Δ*U*/*I*) × *A*, where *ΔU*, *I* and *A* are the step voltage change, current, and contact interfacial area, respectively.

## Author Contributions

**Xiaoqian Wang**: Writing – original draft, data curation, formal analysis, conceptualization, investigation, methodology. **Shulin Bai**: investigation, formal analysis, visualization, validation. **Huiqiang Liang**: validation, visualization. **Rong Liu**: formal analysis, validation, investigation. **Chao Liang**: data curation, methodology, validation. **Yiqing Jia**: investigation, validation. **Xiaokun Feng**: methodology, validation. **Zhenguo Bai**: data curation, validation. **Tian Gao**: validation, visualization. **Huixin Pang**: data curation. **Yongjin Chen**: visualization. **Xin Qian**: methodology, supervision. **Zilu Wang**: formal analysis, writing – review editing. **Haonan Shi**: conceptualization, formal analysis, funding acquisition, writing – review editing. **Sining Wang**: conceptualization, investigation, supervision, funding acquisition, writing – review editing. **Yi Wen**: validation, formal analysis, visualization. **Peikang Bai**: funding acquisition, project administration, resources, writing – review editing. **Lizhong Su**: conceptualization, formal analysis, funding acquisition, investigation, methodology, resources, supervision, writing – review editing. **Li‐Dong Zhao**: conceptualization, formal analysis, supervision, writing – review editing.

## Conflicts of Interest

Lizhong Su, Xiaoqian Wang, and Haonan Shi are inventors on a patent (202511883514.0) submitted by Taiyuan University of Science and Technology that covers the topic of the preparation method for the n‐type Bi2SbSe3 polycrystals.

## Supporting information




**Supporting File**: advs78637‐sup‐0001‐SuppMat.docx.

## Data Availability

The authors declare that the data supporting the findings of this study are available on reasonable request.
