# Supplementary material for: Synthesis Route‐Controlled Atomic Ordering Optimizes Thermoelectric Transport in Bi2SbSe3
Source: Adv Sci (Weinh). 2026 Nov 9:e78637. Online ahead of print. doi: 10.1002/advs.78637 (PMC13749014; doi:10.1002/advs.78637)
Supplement: Supplementary file 1 [file ADVS-9999-e78637-s001.docx]

# Supporting Information

**Synthesis Route-Controlled Atomic Ordering Optimizes Thermoelectric Transport in Bi_2_SbSe_3_**

*Xiaoqian Wang^a†^, Shulin Bai^b†^, Huiqiang Liang^c^, Rong Liu^a^, Chao Liang^a^, Yiqing Jia^a^, Xiaokun Feng^a^, Zhengguo Bai^a^, Tian Gao^b^, Huixin Pang^a^, Yongjin Chen^d^, Xin Qian^c^, Zilu Wang^e^, Haonan Shi^a, f^, Sining Wang^b,g^*, Yi Wen^b^*, Peikang Bai^a, h^*, Lizhong Su^a, f, h^*, Li-Dong Zhao^b^*

^a^School of Materials Science and Engineering, Taiyuan University of Science and Technology, Taiyuan, China.

^b^School of Materials Science and Engineering, Beihang University, Beijing, China.

^c^College of Physics Science and Technology, Hebei University, Baoding, China.

^d^Center for High Pressure Science and Technology Advanced Research (HPSTAR), Beijing, China.

^e^State Key Laboratory of Chemical Biology, Shanghai Institute of Organic Chemistry, University of Chinese Academy of Sciences, Shanghai, China.

^f^State Key Laboratory of Advanced Stainless Steel Material, Taiyuan, China.

^g^School of Materials Science and Technology, China University of Geosciences (Beijing), Beijing, China.

^h^Shanxi Provincial Key Laboratory for Controlled Metal Solidification and Additive Manufacturing, Taiyuan, China.

^†^ These authors contributed equally: Xiaoqian Wang, Shulin Bai.

*Corresponding Author: wangsining@cugb.edu.cn (Sining Wang), ywen23@buaa.edu.cn (Yi Wen), baipeikang@nuc.edu.cn (Peikang Bai), sulizhong@tyust.edu.cn (Lizhong Su).

# 1. Experimental details

1.1 **Thermal conductivity**

The electronic thermal conductivity is calculated based on the Wiedemann-Franz relation, $\text{κ}_{\text{ele}}\text{=}\text{LσT}$, where *e* is the electron charge, and *L* is the Lorentz number estimated using the single parabolic band (SPB) model with acoustic phonon scattering. The following equations provide the calculation details^[1]^:

$$\begin{aligned} L= \left( \frac{k_{B}}{e^{2}} \right)^{2}\left( \frac{\left( r+3.5 \right)F_{r+2.5}\left( \eta\right)}{\left( r+1.5 \right)F_{r+1.5}\left( \eta\right)}-\left[ \frac{\left( r+2.5 \right)F_{r+2.5}\left( \eta\right)}{\left( r+1.5 \right)F_{r+0.5}\left( \eta\right)} \right]^{2} \right)\#\left( S1 \right) \end{aligned}$$

where $\text{k}_{\text{B}}$ is the Boltzmann constant, *e* is the electric charge, *r* is the scattering parameter and $\text{η}$ refers to the reduced Fermi energy ($\text{η }\text{=}{\text{E}_{\text{F}}}/{\text{k}_{\text{B}}\text{T}}$), can be derived from the measured Seebeck coefficients, taking into account acoustic phonon-dominated scattering (*r* = -0.5), based on the following relationship:

$$\begin{aligned} S=\pm\frac{k_{B}}{e}\left[ \frac{\left( r+2.5 \right)F_{r+1.5}\left( \eta\right)}{\left( r+1.5 \right)F_{r+0.5}\left( \eta\right)}-\eta\right],\#\left( S2 \right) \end{aligned}$$

where $\text{F}_{\text{x}}\left( \text{η} \right)$ is Fermi integral

$$\begin{aligned} F_{x}\left( \eta\right)=\int_{0}^{\infty} \frac{x^{n}dx}{1+e^{x-\eta}}.\#\left( S3 \right) \end{aligned}$$

1.2 **Lotgering factor**

The Lotgering factor (*LF*) is defined by the following equation^[2–4]^:

$$\begin{aligned} LF=(P-P_{0})/(1-P_{0})\#\left( S4 \right) \end{aligned}$$

$$\begin{aligned} P_{0}=I_{0}(00l)/\sum I_{0}(hkl)\#\left( \text{S5} \right) \end{aligned}$$

$$\begin{aligned} P=I(00l)/\sum I(hkl)\#\left( \text{S6} \right) \end{aligned}$$

here, *P*_0_ and *P* represent the relative intensities of the preferred orientation planes for the randomly oriented experimental samples, respectively. *I*_0_ (*hkl*) and *I* (*hkl*) represent the diffraction intensities of the (*hkl*) crystal plane for the randomly oriented reference and the experimental sample, respectively.

1.3 **Calculation of weighted mobility (**$\boldsymbol{\mu}_{\boldsymbol{w}}$**)**

The weighted mobility ($\text{μ}_{\text{w}}$) is evaluated by $\text{μ}_{\text{w}}\text{=}\text{μ}\text{ }\left( {\text{m}^{*}}/{\text{m}_{\text{e}}} \right)^{\text{3}/\text{2}}$, where $\text{m}_{\text{e}}$ denotes the unit mass of an electron. With measured Seebeck coefficient and electrical conductivity, and applying equations S2 and S3, the $\text{μ}_{\text{w}}$ could be obtained through the following calculation^[5]^:

$$\begin{aligned} \mu_{w}=\frac{3\sigma}{8\pi F_{0}\left( \eta\right)}\left( \frac{h^{2}}{2m_{e}k_{B}T} \right)^{1.5}\#\left( S7 \right) \end{aligned}$$

1.4 **Estimation of electron mean free path**

The electron mean free paths (*l*_e_) of ordered and disordered Bi_2_SbSe_3_ were estimated from the room-temperature Hall data using the following equations^[6]^:

$$\begin{aligned} l_{e}=v_{F}\tau\#\left( \text{S8} \right) \end{aligned}$$

$$\begin{aligned} v_{F}=\frac{\hbar k_{F}}{m^{*}}, \tau=\frac{\mu m^{*}}{e}\#\left( \text{S}\text{9} \right) \end{aligned}$$

$$\begin{aligned} k_{F}={(3\pi^{2}n)}^{1/3}\#\left( \text{S10} \right) \end{aligned}$$

where *v*_F_ is the Fermi velocity, *τ* is the carrier relaxation time, *ħ* is the reduced Planck constant, *k*_F_ is the Fermi wavevector, *m*^*^ is the effective mass, *n* is the carrier concentration, *μ* is the carrier mobility, and *e* is the elementary charge.

1.5 **Density functional theory (DFT) calculations**

DFT calculations were performed using the projector-augmented wave (PAW) method implemented in the Vienna Ab-initio Simulation Package (VASP)^[7–9]^. Because Bi and Sb exhibit mixed fractional occupancies on the cation sites of Bi_2_SbSe_3_, such occupational disorder cannot be directly represented in the periodic structural models employed in the present conventional DFT calculations. Therefore, averaged and approximated structural models based on the BiSe crystal framework were adopted. For the ordered and disordered samples, the BiSe structural models were separately refined using the corresponding SR-XRD data collected at 300 K, from which the averaged lattice parameters and atomic positions were obtained. These experimentally derived structural parameters were then used to construct the respective input structures for the DFT calculations. Accordingly, the calculated models do not explicitly reproduce the microscopic Bi/Sb occupational configurations, but instead provide averaged structural representations of the experimentally observed ordered and disordered states. The calculations were therefore used to evaluate the influence of the structural changes associated with different atomic-ordering states on the electronic band structure. The calculated projected density of states are shown in **Figure S12**, and the longitudinal and shear sound velocities are also calculated (**Table S3**).

1.6 **Acoustic velocity measurements**

The longitudinal and shear sound velocities were measured using a pulser/receiver (CTS-8077PR, SIUI, China) in combination with an oscilloscope (TBS1102C, Tektronix, China). They can be derived from the thickness of the sample *d* and travel time Δ*t*, and the uncertainty of acoustic velocities is estimated to be within 5%.

1.7 **Elastic and thermodynamic properties**

In this work, average sound velocity (*ν*_a_), Poisson ratio (*ν*_p_), and Grüneisen parameter (*γ*) can be calculated from the sound velocity as follows^[10–15]^:

$$\begin{aligned} \nu_{a}=\left[ \frac{1}{3}\left( \frac{1}{{\nu_{l}}^{3}}+\frac{2}{\nu_{s}} \right) \right]^{-1/3}\#\left( S11 \right) \end{aligned}$$

$$\begin{aligned} \nu_{p}=\frac{1-2\left( {\nu_{s}}/{\nu_{l}} \right)^{2}}{2-2\left( {\nu_{s}}/{\nu_{l}} \right)^{2}}\#\left( S12 \right) \end{aligned}$$

$$\begin{aligned} \gamma=\frac{3}{2}\left( \frac{1+\nu_{p}}{2-3\nu_{p}} \right) \#\left( S13 \right) \end{aligned}$$

where *ν*_l_ and *ν*_s_ denote longitudinal and shear sound velocities, respectively. The sound velocity can be acquired directly in the ultrasonic pulse echo measurements (Table S4); the value of the Debye temperature also reflects the thermal conductivity to some extent. Debye temperature *θ*_D_ can be estimated as follows^[10,16]^:

$$\begin{aligned} \theta_{D}=\frac{h}{k_{B}}\left[ \frac{3N}{4\pi V} \right]^{1/3}\nu_{a} \#\left( S14 \right) \end{aligned}$$

1.8 **The preparation process of thermoelectric device particles**

1. Disordered Bi_2_SbSe_3_ wafers were cut parallel to the pressure direction into 3 mm-thick slices and further processed into 3 × 3 × 9 mm^3^ rectangular bars.
2. The top and bottom surfaces were sequentially polished using 1000-, 2000-, and 3000-grit SiC abrasive paper to ensure smooth contact interfaces.
3. The samples were ultrasonically cleaned to remove surface debris.
4. Surface treatment was carried out by immersing the samples in a hydrochloric acid/deionized water solution (1:1 volume ratio), followed by ultrasonic cleaning to remove residual acid.
5. Both surfaces were coated with a Ni layer via electroless plating (plating solution: Q/YS.602-2).
6. Sn_96.5_Ag_3.0_Cu_0.5_ foil was used as solder for both cold and hot ends, and electrical contacts were formed using a reflow soldering process (Shandong Puhui Technology Co., Ltd.).
   1.9 **Single-leg power generation efficiency test**

We tested the single-leg module using the TE-X-MS (designed by Xi'an Jiaotong University). This equipment provides the temperature difference Δ*T* across both ends of the single-leg module and the resulting voltage Δ*U*. The voltage Δ*U* determined by the temperature difference forms the temperature difference value *S*. When current *I* is applied, the current dependence of power output *P* is as follows:

$$\begin{aligned} P=I\left( \Delta U-IR \right)=-RI^{2}+S\Delta TI\#\left( S15 \right) \end{aligned}$$

During the measurement process, the maximum power output Pmax can be calculated based on the current dependence relationship. Here, the resistance *R* represents the internal resistance value. This instrument employs a flow meter to measure heat flux. The relationship between the temperature difference between the inlet temperature Tin and the outlet temperature Tout of the liquid in the flow meter and the liquid flow velocity *v*, as well as the module heat flux, is expressed by the following equation:

$$\begin{aligned} T_{out}-T_{in}=\frac{Q}{C}\frac{1}{v}\#\left( S16 \right) \end{aligned}$$

In the equation, *C* represents the specific heat capacity per unit volume. Within the analysis software, heat flux can be calculated using the rate of change in temperature difference between the liquid inlet and outlet temperatures, along with the flow velocity. The relationship between heat flux *Q* and conversion efficiency *η* for the thermoelectric module is expressed as follows:

$$\begin{aligned} \eta=\frac{P}{Q+P}\#\left( S17 \right) \end{aligned}$$

This heat flow comprises the heat *Q*_0_ generated by the temperature difference across the sample and the amount of Joule heat produced, expressed as:

$\begin{aligned} Q=Q\left( 0 \right)+ST_{C}I+\frac{1}{2}I^{2}R\#\left( S18 \right) \end{aligned}$The sample temperature at the low-temperature end is denoted as *T*_C_. The conversion efficiency is calculated using the following formula:

$$\begin{aligned} \eta=\frac{RI^{2}+S\left( T_{H}-T_{C} \right)I}{-\frac{1}{2}RI^{2}+ST_{H}I+Q\left( 0 \right)}\#\left( S19 \right) \end{aligned}$$

# 2. Tables

**Table S1.** Densities of Bi_1-x_Sb_x_Se and disordered Bi_2_SbSe_3_ samples.

| Samples | Density (g cm^-3^) |
| --- | --- |
| ordered Bi_2_SbSe_3_ | 7.4042 |
| disordered Bi_2_SbSe_3_ | 7.1807 |
| BiSe | 7.9592 |
| Bi_0.9_Sb_0.1_Se | 7.8843 |
| Bi_0.8_Sb_0.2_Se | 7.5313 |
| Bi_0.6_Sb_0.4_Se | 7.2371 |
| disordered Bi_2_SbSe_3_-weak texture (*LF*=0.0134)*‖* | 7.2533 |
| disordered Bi_2_SbSe_3_-weak texture (*LF*=0.1176)*‖* | 7.2745 |

**Table S2.** The average intensity of (Bi/Sb)_1_ and (Bi/Sb)_2_ sites.

| Samples | Intensity at (Bi/Sb)_1_ site | Intensity at (Bi/Sb)_2_ site |
| --- | --- | --- |
| ordered Bi_2_SbSe_3_ | 10.4795 | 7.5679 |
| disordered Bi_2_SbSe_3_ | 11.0528 | 8.7836 |

**Table S3.** Calculated shear sound velocity (*ν*_s_), longitudinal sound velocity (*ν*_l_), average sound velocity (*ν*_a_), Poisson ratio (*ν*_p_), Grüneisen parameter (*γ*) and Debye temperature (*θ*_D_) for ordered Bi_2_SbSe_3_ and disordered Bi_2_SbSe_3_ from band structure.

| Samples | *ν*_s_ (m s^-1^) | *ν*_l_ (m s^-1^) | *ν*_a_ (m s^-1^) | *ν*_p_ | *γ* | *θ*_D_ (K) |  |
| --- | --- | --- | --- | --- | --- | --- | --- |
| ordered Bi_2_SbSe_3_ | 1365.54 | 2883.74 | 1536.43 | 0.36 | 2.18 | 150.03 | |
| disordered Bi_2_SbSe_3_ | 1336.27 | 2843.00 | 1504.05 | 0.36 | 2.20 | 147.34 | |

**Table S4.** Experimental *ν*_s_, *ν*_l_, *ν*_a_, *ν*_p_, *γ* and *θ*_D_ for ordered Bi_2_SbSe_3_ and disordered Bi_2_SbSe_3_.

| Samples | *ν*_s_ (m s^-1^) | *ν*_l_ (m s^-1^) | *ν*_a_ (m s^-1^) | *ν*_p_ | *γ* | *θ*_D_ (K) |
| --- | --- | --- | --- | --- | --- | --- |
| ordered Bi_2_SbSe_3_ | 1647.00 | 2838.00 | 1827.65 | 0.25 | 1.48 | 178.46 |
| disordered Bi_2_SbSe_3_ | 1639.00 | 2777.00 | 1815.98 | 0.23 | 1.42 | 177.90 |

# 3. Supplementary Figures

**Figure S1.** (A) Powder X-ray diffraction (XRD) patterns of Bi_1-x_Sb_x_Se. (B) Enlarged views of the 25–50°.

**Figure S2.** The thermoelectric properties for Bi_1-x_Sb_x_Se parallel to the press direction of SPS. (A) Electrical conductivity. (B) Seebeck coefficient. (C) Power factor. (D) Lorenz number. (E) Thermal diffusivity. (F) Total thermal conductivity. (G) Electronic thermal conductivity. (H) Lattice thermal conductivity. (I) *ZT* value.

**Figure S3.** The thermoelectric properties for Bi_1-x_Sb_x_Se perpendicular to the press direction of SPS. (A) Electrical conductivity. (B) Seebeck coefficient. (C) Power factor. (D) Lorenz number. (E) Thermal diffusivity. (F) Total thermal conductivity. (G) Electronic thermal conductivity. (H) Lattice thermal conductivity. (I) *ZT* value.

**Figure S4.** XRD patterns of disordered Bi_2_SbSe_3_ samples before and after texture regulation. (A) Perpendicular and (B) parallel to the SPS pressing directions.

**Figure S5.** Thermoelectric properties of disordered Bi_2_SbSe_3_-weak texture (*LF*=0.0134). (A) Electrical conductivity. (B) Seebeck coefficient. (C) Power factor. (D) Lorenz number. (E) Thermal diffusivity. (F) Total thermal conductivity. (G) Electronic thermal conductivity. (H) Lattice thermal conductivity. (I) *ZT* value.

**Figure S6.** Thermoelectric properties of disordered Bi_2_SbSe_3_-weak texture (*LF*=0.1176). (A) Electrical conductivity. (B) Seebeck coefficient. (C) Power factor. (D) Lorenz number. (E) Thermal diffusivity. (F) Total thermal conductivity. (G) Electronic thermal conductivity. (H) Lattice thermal conductivity. (I) *ZT* value.

**Figure S7.** Thermoelectric properties of disordered Bi_2_SbSe_3_ perpendicular to the press direction of SPS. (A) Electrical conductivity. (B) Seebeck coefficient. (C) Power factor. (D) Lorenz number. (E) Thermal diffusivity. (F) Total thermal conductivity. (G) Electronic thermal conductivity. (H) Lattice thermal conductivity. (I) *ZT* value.

**Figure S8.** The bulk XRD patterns of ordered and disordered Bi_2_SbSe_3_ samples perpendicular to the SPS pressing direction.

**Figure S9**. Microstructure observation of ordered Bi_2_SbSe_3_. (A) SEM image acquired in secondary electron (SE) mode and (B)-(D) corresponding EDS elemental maps of Bi, Se, and Sb, respectively. (E) Electron image of the Ar-ion polished sample and (F)-(H) corresponding EDS elemental maps of Bi, Se, and Sb, respectively, obtained after Ar-ion polishing. (I) EDS spectrum of ordered Bi_2_SbSe_3_.

**Figure S10**. Microstructure observation of disordered Bi_2_SbSe_3_. (A) SEM image acquired in secondary electron (SE) mode and (B)-(D) corresponding EDS elemental maps of Bi, Se, and Sb, respectively. (E) Electron image of the Ar-ion polished sample and (F)-(H) corresponding EDS elemental maps of Bi, Se, and Sb, respectively, obtained after Ar-ion polishing. (I) EDS spectrum of disordered Bi_2_SbSe_3_.

**Figure S11.** Temperature-dependent SR-XRD patterns of disordered Bi_2_SbSe_3_.

**Figure S12.** Projected density of states (PDOS) of (A) ordered Bi_2_SbSe_3_ and (B) disordered Bi_2_SbSe_3._

**Figure S13.** Electron backscatter diffraction (EBSD) characterization of ordered Bi_2_SbSe_3_. (A) Band contrast map and (B) corresponding grain size distribution. The average grain size is approximately 3.1 μm. (C) Kernel average misorientation (KAM) map with grain boundaries.

**Figure S14.** EBSD characterization of disordered Bi_2_SbSe_3_. (A) Band contrast map and (B) corresponding grain size distribution. The average grain size is approximately 3.9 μm. (C) KAM map with grain boundaries.

**Figure S15.** Thermal transport properties parallel to the SPS pressing direction. (A) Electronic thermal conductivity. (B) Thermal diffusivity.

**Figure S16.** Quality factor *B* parallel to the SPS pressing direction*.*

**Figure S17.** Performance of the single-leg device based on disordered Bi_2_SbSe_3_ at different hot-side temperatures. (A) Output voltage (*U*). (B) Output power (*P*). (C) Heat flux (*Q*).

**Figure S18**. Single-leg device performance of disordered Bi_2_SbSe_3_. (A) Experimental and theoretical conversion efficiencies as a function of temperature difference. (B) Electrical contact resistance measurement.

**Figure S19.** Electrical transport properties of disordered Bi_2_SbSe_3_ during repeated heating and cooling cycles at target temperatures of 523, 573, and 623 K. (A) Electrical conductivity. (B) Seebeck coefficient. (C) Power factor.

# Supplementary Reference

1. W. He, D. Wang, H. Wu, et al., “High Thermoelectric Performance in Low-Cost SnS_0.91_Se_0.09_ Crystals,” *Science* *365*, no. 6460 (2019): 1418–1424, https://doi.org/10.1126/science.aax5123.

2. M. Zhang, C. Zhang, Y. You, et al., “Electron Density Optimization and the Anisotropic Thermoelectric Properties of Ti Self-Intercalated Ti_1+_ *_x_* S_2_ Compounds,” *ACS Applied Materials & Interfaces* *10*, no. 38 (2018): 32344–32354, https://doi.org/10.1021/acsami.8b10449.

3. R. Furushima, S. Tanaka, Z. Kato, and K. Uematsu, “Orientation Distribution-Lotgering Factor Relationship in a Polycrystalline Material-as an Example of Bismuth Titanate Prepared by a Magnetic Field,” *Journal of the Ceramic Society of Japan* *118*, no. 1382 (2010): 921–926, https://doi.org/10.2109/jcersj2.118.921.

4. J.-J. Shen, T.-J. Zhu, X.-B. Zhao, S.-N. Zhang, S.-H. Yang, and Z.-Z. Yin, “Recrystallization Induced in Situ Nanostructures in Bulk Bismuth Antimony Tellurides: A Simple Top down Route and Improved Thermoelectric Properties,” *Energy & Environmental Science* *3*, no. 10 (2010): 1519, https://doi.org/10.1039/c0ee00012d.

5. G. J. Snyder, A. H. Snyder, M. Wood, R. Gurunathan, B. H. Snyder, and C. Niu, “Weighted Mobility,” *Advanced Materials* *32*, no. 25 (2020): 2001537, https://doi.org/10.1002/adma.202001537.

6. W. Zheng, H. Ramsden, S. Ippolito, et al., “Photothermal Effects Control Ultrafast Charge Transport in Titanium Carbide MXenes,” *Nature Communications* *17*, no. 1 (2026): 1201, https://doi.org/10.1038/s41467-026-68831-4.

7. P. E. Blöchl, “Projector Augmented-Wave Method,” *Physical Review B* *50*, no. 24 (1994): 17953–17979, https://doi.org/10.1103/PhysRevB.50.17953.

8. G. Kresse, and D. Joubert, “From Ultrasoft Pseudopotentials to the Projector Augmented-Wave Method,” *Physical Review B* *59*, no. 3 (1999): 1758–1775, https://doi.org/10.1103/PhysRevB.59.1758.

9. G. Kresse, and J. Furthmüller, “Efficient Iterative Schemes for *Ab Initio* Total-Energy Calculations Using a Plane-Wave Basis Set,” *Physical Review B* *54*, no. 16 (1996): 11169–11186, https://doi.org/10.1103/PhysRevB.54.11169.

10. K. Kurosaki, A. Kosuga, H. Muta, M. Uno, and S. Yamanaka, “Ag_9_TlTe_5_: A High-Performance Thermoelectric Bulk Material with Extremely Low Thermal Conductivity,” *Applied Physics Letters* *87*, no. 6 (2005): 061919, https://doi.org/10.1063/1.2009828.

11. Y. Xiao, C. Chang, Y. Pei, et al., “Origin of Low Thermal Conductivity in SnSe,” *Physical Review B* *94*, no. 12 (2016): 125203, https://doi.org/10.1103/PhysRevB.94.125203.

12. C. L. Wan, W. Pan, Q. Xu, et al., “Effect of Point Defects on the Thermal Transport Properties of (La_x_Gd_1−x_)_2_Zr_2_O_7_: Experiment and Theoretical Model,” *Physical Review B* *74*, no. 14 (2006): 144109, https://doi.org/10.1103/PhysRevB.74.144109.

13. Y.-L. Pei, J. He, J.-F. Li, et al., “High Thermoelectric Performance of Oxyselenides: Intrinsically Low Thermal Conductivity of Ca-Doped BiCuSeO,” *NPG Asia Materials* *5*, no. 5 (2013): e47–e47, https://doi.org/10.1038/am.2013.15.

14. C. Wan, Z. Qu, Y. He, D. Luan, and W. Pan, “Ultralow Thermal Conductivity in Highly Anion-Defective Aluminates,” *Physical Review Letters* *101*, no. 8 (2008): 085901, https://doi.org/10.1103/PhysRevLett.101.085901.

15. J. Y. Cho, X. Shi, J. R. Salvador, et al., “Thermoelectric Properties and Investigations of Low Thermal Conductivity in Ga-Doped Cu_2_GeSe_3_,” *Physical Review B* *84*, no. 8 (2011): 085207, https://doi.org/10.1103/PhysRevB.84.085207.

16. D. T. Morelli, V. Jovovic, and J. P. Heremans, “Intrinsically Minimal Thermal Conductivity in Cubic I−V−VI_2_ Semiconductors,” *Physical Review Letters* *101*, no. 3 (2008): 035901, https://doi.org/10.1103/PhysRevLett.101.035901.
